# Characterization of Gut Microbiome Composition in Patients with Triple-Negative Breast Cancer Treated with Neoadjuvant Chemotherapy

**DOI:** 10.1093/oncolo/oyad060

**Published:** 2023-03-20

**Authors:** Grazia Vernaci, Edoardo Vincenzo Savarino, Ilaria Patuzzi, Sonia Facchin, Fabiana Zingone, Davide Massa, Giovanni Faggioni, Tommaso Giarratano, Federica Miglietta, Gaia Griguolo, Matteo Fassan, Marcello Lo Mele, Elisa Gasparini, Giancarlo Bisagni, Valentina Guarneri, Maria Vittoria Dieci

**Affiliations:** Medical Oncology 2, Veneto Institute of Oncology IOV-IRCCS, Padova, Italy; Gastroenterology Unit, Department of Surgery, Oncology and Gastroenterology, University of Padua, Padua, Italy; Gastroenterology Unit, Azienda Ospedale Università di Padova, Padova, Italy; Research & Development, Eubiome Srl, Padova, Italy; Gastroenterology Unit, Department of Surgery, Oncology and Gastroenterology, University of Padua, Padua, Italy; Gastroenterology Unit, Azienda Ospedale Università di Padova, Padova, Italy; Gastroenterology Unit, Department of Surgery, Oncology and Gastroenterology, University of Padua, Padua, Italy; Gastroenterology Unit, Azienda Ospedale Università di Padova, Padova, Italy; Medical Oncology 2, Veneto Institute of Oncology IOV-IRCCS, Padova, Italy; Department of Surgery, Oncology and Gastroenterology, University of Padova, Padova, Italy; Medical Oncology 2, Veneto Institute of Oncology IOV-IRCCS, Padova, Italy; Medical Oncology 2, Veneto Institute of Oncology IOV-IRCCS, Padova, Italy; Medical Oncology 2, Veneto Institute of Oncology IOV-IRCCS, Padova, Italy; Department of Surgery, Oncology and Gastroenterology, University of Padova, Padova, Italy; Medical Oncology 2, Veneto Institute of Oncology IOV-IRCCS, Padova, Italy; Department of Surgery, Oncology and Gastroenterology, University of Padova, Padova, Italy; Department of Medicine, Surgical Pathology Unit, University of Padua, Padua, Italy; Veneto Institute of Oncology, IOV-IRCCS, Padua, Italy; Department of Pathology, Azienda Ospedale Università Padova, Padova, Italy; Breast Cancer Unit, Azienda Unità Sanitaria Locale-IRCCS di Reggio Emilia, Reggio Emilia, Italy; Oncology Unit, Azienda Unità Sanitaria Locale-IRCCS di Reggio Emilia, Reggio Emilia, Italy; Breast Cancer Unit, Azienda Unità Sanitaria Locale-IRCCS di Reggio Emilia, Reggio Emilia, Italy; Oncology Unit, Azienda Unità Sanitaria Locale-IRCCS di Reggio Emilia, Reggio Emilia, Italy; Medical Oncology 2, Veneto Institute of Oncology IOV-IRCCS, Padova, Italy; Department of Surgery, Oncology and Gastroenterology, University of Padova, Padova, Italy; Medical Oncology 2, Veneto Institute of Oncology IOV-IRCCS, Padova, Italy; Department of Surgery, Oncology and Gastroenterology, University of Padova, Padova, Italy

**Keywords:** microbiome, breast cancer, TNBC

## Abstract

**Introduction:**

Patients with triple-negative breast cancer (TNBC) achieving a pathological complete response (pCR) after neoadjuvant chemotherapy have a better event-free survival. The role of gut microbiome in early TNBC is underexplored.

**Methods:**

Microbiome was analyzed by 16SrRNA sequencing.

**Results:**

Twenty-five patients with TNBC treated with neoadjuvant anthracycline/taxane-based chemotherapy were included. Fifty-six percent achieved a pCR. Fecal samples were collected before (t0), at 1 (t1), and 8 weeks (t2) from chemotherapy. Overall, 68/75 samples (90.7%) were suitable for microbiome analysis. At t0, pCR group showed a significantly higher α-diversity as compared with no-pCR, (*P = .*049). The PERMANOVA test on β-diversity highlighted a significant difference in terms of BMI (*P* = 0.039). Among patients with available matched samples at t0 and t1, no significant variation in microbiome composition was reported over time.

**Conclusions:**

Fecal microbiome analysis in early TNBC is feasible and deserves further investigation in order to unravel its complex correlation with immunity and cancer.

## Teaser

The gut microbiome has gained attention as a potential biomarker in patients with cancer; however, little is known about the role of gut microbiome in modulating response to standard neoadjuvant chemotherapy in patients diagnosed with early stage triple-negative breast cancer (TNBC). This article assesses the feasibility of gut microbiome analysis in patients undergoing neoadjuvant chemotherapy for early stage TNBC, evaluates its impact on treatment response and association with clinicopathologic factors, and describes longitudinal changes before and after exposure to chemotherapy.

**Implications for Practice**

Compelling evidence suggests how gut microbiome may influence the effectiveness of chemotherapy (CT) and immune-checkpoint inhibitors. Its role in modulating response to neoadjuvant CT in patients with triple-negative breast cancer (TNBC) is underexplored. In this study we demonstrated the feasibility of gut microbiome analysis in this population. Furthermore, patients achieving a pathological complete response had a higher a-diversity, and a trend to a higher richness as compared with those with residual disease. Gut microbiome composition was stable over time, suggesting how its modulation at the baseline could affect treatment effectiveness.

## Introduction

Breast cancer (BC) is the most common malignancy and the leading cause of death in women worldwide. Triple-negative breast cancer (TNBC), accounting for approximately 15%-20% of all invasive breast tumors, is the most aggressive subtype and chemotherapy is the mainstay of treatment in both early and advanced stages.^1^ Achieving a pathological complete response (pCR) after neoadjuvant chemotherapy (NACT) is considered a valid surrogate for long-term outcome at the patient level.^2^

The crucial role of the immune system in TNBC prognosis and response to treatments has been previously demonstrated.^3-5^ For instance, the level of tumor-infiltrating lymphocytes (TILs) reached level Ib evidence as a predictive biomarker for pCR after NACT in early patients with TNBC.^3-5^

The trillions of bacteria that colonize the human gut, known as microbiota, can affect both the host’s innate and adaptive immune system in several ways.^6^ In fact, the state of dysbiosis has been related to many pathological conditions, including cancer.^7^ On the other hand, an intact gut microbiota has been shown to promote tumor immunosurveillance.^8^ Furthermore, various studies showed that the gut microbiome composition may influence the effectiveness of conventional chemotherapy and Immune-Checkpoint Inhibitors (ICIs) in several solid tumors (i.e. melanoma, NSCLC, HCC), in both clinical and preclinical models.^8-14^ A recent study pointed out that gut microbiome can impact BC prognosis in humans and mice.^15^ In particular, metagenomic stool composition was correlated with clinical prognostic parameters (such as tumor size, nodal status, grade and staging) in early patients with BC. Furthermore, by using avatar mouse models receiving fecal microbial transplantation, 2 groups of stool influencing the pattern of tumor progression and chemotherapy efficacy were identified. In patients with BC, chemotherapy was also able to induce a shift in microbiome composition, favoring the colonization of health-­related bacteria and the decrease of species associated with the aggressiveness of the disease.^15^

Given this evidence, during the past decade the gut microbiome has drawn researchers’ attention as a potential biomarker in patients with cancer. However, little is still known about the role of gut microbiome in modulating response to standard neoadjuvant chemotherapy in patients diagnosed with early TNBC.

In this study, we aimed at: i) assessing the feasibility of gut microbiome analysis in patients undergoing neoadjuvant chemotherapy for early TNBC, ii) evaluating its impact on treatment response and association with clinicopathologic factors, and iii) describing longitudinal changes before and after exposure to chemotherapy.

## Results

### Patients’ Population

The characteristics of the 25 patients included in the present analysis are described in Table 1.

**Table 1.** Patient’s characteristics.

Characteristic	*n* (%)^a^
Age (median, range)	
Menopause	52 (45-58)
Yes	14 (56)
No	11 (44)
BMI	
≥25	10 (40)
<25	15 (60)
cN	
Neg	9 (36)
Pos	16 (64)
Stage	
I	3 (12)
II	18 (72)
III	4 (16)
TILs biopsy (median, range)	
CT type	30 (13.75-46.25)
Anthra + Taxane	2 (8)
Anthra + Taxane+ Platinum	23 (92)
TILs surgery (median, range)	
pCR	5 (5-15)
Yes	14 (56)
No	11 (44)

^a^Data shown is presented as n (%) unless otherwise indicated.

Abbreviations: BMI, body mass index; TILs, tumor-infiltrating lymphocytes; pCR, pathological complete response.

Median age was 52 years (range 45-58). Fourteen out of 25 patients (56%) were postmenopausal at the time of BC diagnosis. Ten patients (40%) were overweight or obese. The majority of the patients (88%, *n* = 22) had a clinical stages II-III tumor. Nodal involvement was present in more than half of the patients at the diagnosis of BC (64%, *n* = 16). All patients were candidate to a sequential neoadjuvant regimen including paclitaxel administered weekly for 12 weeks followed by epirubicin/cyclophosphamide for 4 courses every 3 weeks. Carboplatin (weekly, AUC2) was also added to the taxane segment for 23 patients (92%). Five patients (20%) failed to complete all the planned chemotherapy administrations due to toxicity. Fourteen (56%) patients achieved a pCR.

Median TILs on the diagnostic core biopsy were 30% (range 13.75-46.25). TILs were evaluable in 9 out of 11 samples from patients with residual disease after NACT. Median TILs level on residual disease was 5% (range 5-15).

### Feasibility of Gut Microbiome Analysis

Seventy-two of the 75 samples expected in the study design were collected (Supplementary Fig. S1). A total of 4,743,112 raw reads were obtained from the sequencing of the 72 samples, with a median frequency per sample of 67,026.5. After preprocessing and ASV table construction, 2,848,932 reads were retained for further analyses, with a median frequency per sample of 42,268.5. Four samples (2 at t0 and 2 at t1) were then excluded from downstream analyses as they presented too few reads, with a final number of 68 samples suitable for proper microbiome analysis. The primary feasibility endpoint of the study was met, with 68 samples collected and technically evaluable for microbiome analysis out of 75 expected: 90.7% with a 95% CI excluding 80% (82.5%-95.7%). The final list of samples included in the following part of the analysis is reported in Table 2, together with the detail of the final sequences per samples.

**Table 2.** List of samples included in the final analysis.

Patient ID	Reads per timepoint		
	t0	t1	t2
P1	55,415	54,143	53,724
P2	50,342	54,982	40,711
P3	52,868	57,518	42,967
P4	50,660	55,264	46,055
P5	37,361	18,255	47,194
P6	29,388	6444	42,585
P7	42,578	50,305	58,873
P8	40,877	46,536	43,382
P9	13,852	58,373	51,420
P10	27,807	27,512	25,492
P11	44,340	48,154	41,055
P12	35,896	31,613	28,890
P13	45,067	37,184	32,846
P14	55,596	34,777	46,083
P15	41,959	X	21,564
P16	47,281	41176	36,179
P17	45,247	65,361	45,119
P18	27,491	NC	NC
P19	X	X	40,520
P20	X	59,556	59,273
P21	24,983	73,709	62,535
P22	34,695	6499	48,705
P23	23,094	37,538	55,160
P24	22,834	29,023	35,754
P25	40,858	NC	58,409

Considering the known potential effects on gut microbiome, we decided to further exclude samples collected within 90 days of antibiotic therapy (1 t0 sample, 7 t1 samples, 15 t2 samples).

CONSORT diagram is shown in Supplementary Fig. S1.

### Gut Microbiome Composition at Baseline: Association with Clinicopathologic Factors and pCR

**Figure 1.**
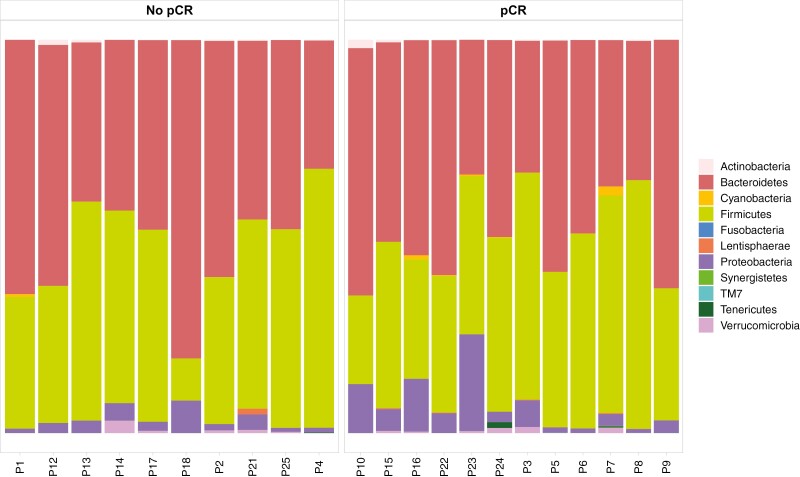
Bar plot showing phyla distribution in the total population.

The taxonomic profiling of baseline stool samples revealed that Bacteroidetes and Firmicutes were the most abundant phyla (Fig. 1).

In order to assess the association between microbiome composition and response to treatment, we grouped patients into 2 categories according to whether they had achieved a pCR (pCR group) or not (no-pCR group).

**Figure 2.**
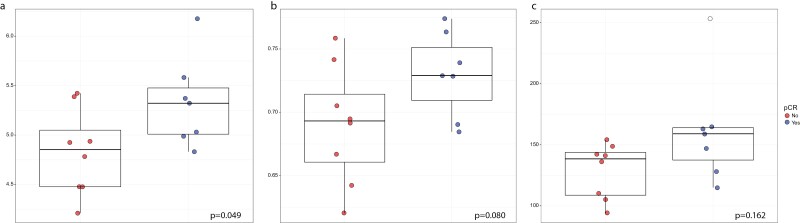
Box plot of baseline α-diversity between pCR and no-pCR patients at the ASV level according to Shannon Index (**a**), Pielou Eveness (**b**), and Richness (**c**). Outlier values are depicted in white and were excluded from the statistical test. Abbreviations: pCR, pathological complete response; ASV, amplicon sequence variant.

After applying the rarefaction threshold, 4 samples were excluded from α-diversity evaluation at the baseline. The Dixon test (Supplementary Tables S1 and S2) highlighted the presence of one outlier value for Richness, both at ASV and species level. This value was consequently excluded from the related statistical testing. Considering amplicon sequence variant (ASV), the pCR group had a significantly higher α-diversity evaluated by using Shannon diversity index (*P* = 0.049) (Fig. 2a). Furthermore, we observed a difference between the 2 groups, although not statistically significant, in terms of Pielou eveness, and a trend toward a higher richness in pCR patients (*P*–value, respectively, 0.080 and 0.162, Fig. 2b and 2c). α-Diversity at the species level did not significantly differ between the 2 groups (Supplementary Fig. S2). No significant correlation between α-diversity and clinicopathological features was found at any level.

**Figure 3.**
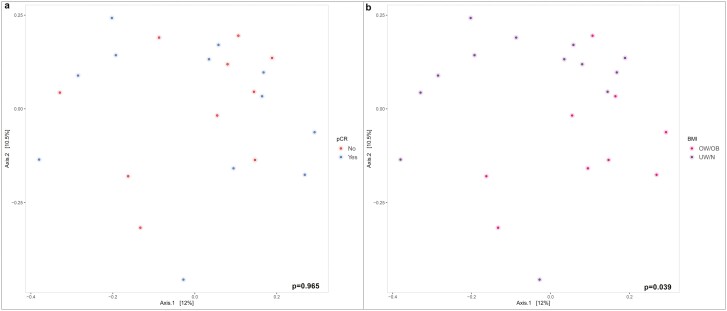
Plot of Bray-Curtis β-diversity Index at the baseline in terms of ASV according to pCR (**a**) and BMI as a binary variable (**b**). Abbreviations: ASV, amplicon sequence variant; BMI, body mass index; pCR, pathological complete response.

At the ASV level, The PERMANOVA test performed on Bray-Curtis β-diversity at the baseline showed the absence of significant differences between pCR and no-pCR patients (*P* = 0.965; Fig. 3a). Considering the main clinicopathological features, a statistically significant difference was highlighted in terms of BMI, considered as a binary variable (overweight/obese vs normal/underweight; Bray-Curtis, *P* = 0.039 [Fig. 3b]). The PERMANOVA test performed on Weighted Unifrac β-diversity revealed a significant difference between pre- and post-menopausal patients (*P* = 0.035, not shown).

When evaluating the differential abundance of bacterial species, *Bacteroides eggerthii* was differentially abundant in the 2 groups of patients (mean 0.00%, range 0.00-0.01, SD 0.00% and mean 1.44%, range 0.00-5.81, SD 2.37%, respectively).

### Gut Microbiome Composition: Longitudinal Changes

As the majority of the patients had received antibiotic therapy during the course of neoadjuvant treatment, and considering the known potential effects on gut microbiome, we decided to exclude t1 and t2 samples collected within 90 days from the last antibiotics administration, retaining 14 t1 and 9 t2 samples. Thus, 14 patients had evaluable fecal samples for both t0 and t1. Seven patients had evaluable fecal samples for all 3 consecutive timepoints. Due to the limited sample size, we conducted only descriptive analyses without applying any statistical test.

**Figure 4.**
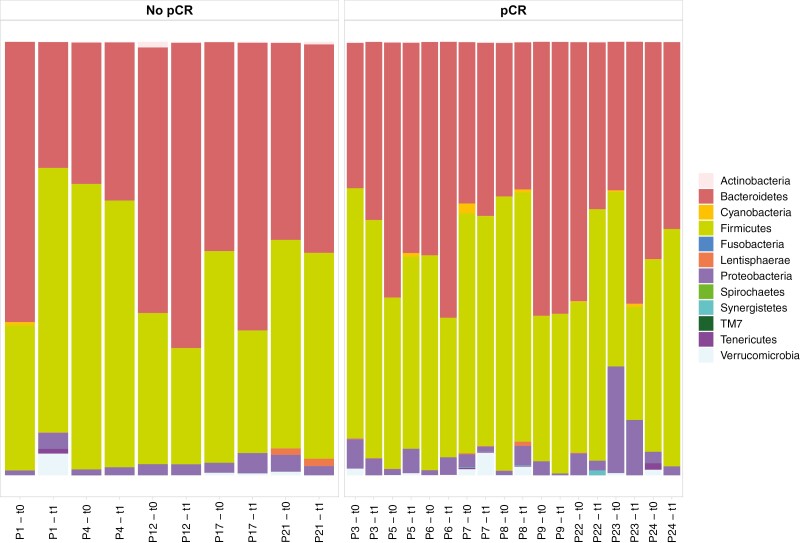
Bar plot showing phyla distribution in matched samples before (t0) and after chemotherapy (t1). Only patients with available consecutive samples for both t0 and t1 were included.

**Figure 5.**
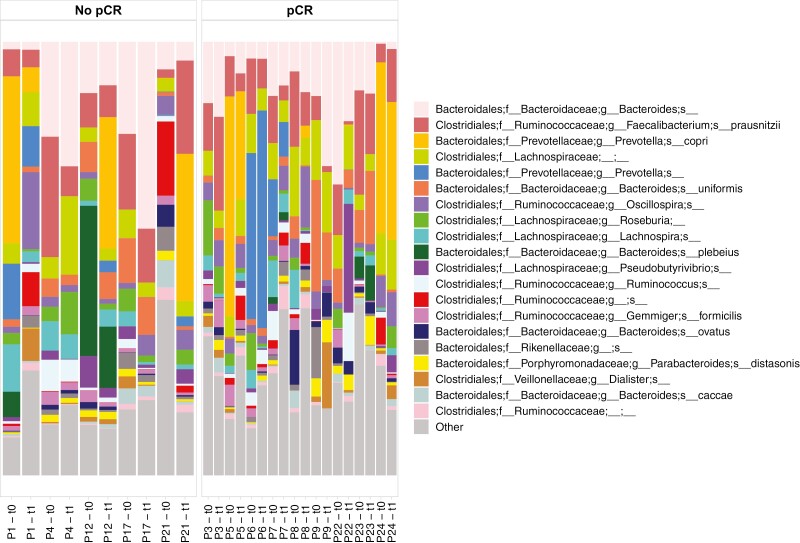
Bar plot showing the 20 most represented species in matched samples before (t0) and after chemotherapy (t1). Only patients with available consecutive samples for both t0 and t1 were included.

Composition at the phyla and species levels remained substantially stable across the different timepoints for each patient, in both the pCR and no-pCR groups (Figs 4 and 5).

There was no apparent change in median Firmicutes/Bacteroidetes (F/B) ratio after chemotherapy in both the pCR and no-pCR groups (Supplementary Fig. S3).

Species composition also remained substantially stable over time.

## Discussion

In the last decade, the gut microbiome composition has been associated with different clinical conditions, including various forms of cancer, in terms of pathogenesis, diagnosis and therapeutic outcome.^16^ However, evidence is limited regarding its role in modulating response to standard neoadjuvant chemotherapy in patients diagnosed with early TNBC. Thus, this study represents one of the first experiences evaluating the role of gut microbiome composition in patients with TNBC treated with neoadjuvant chemotherapy. First, we demonstrated the feasibility of gut microbiome analysis in this population. In terms of microbial composition of stool samples collected before treatment, Firmicutes and Bacteroidetes were the most abundant phyla, as expected, in both pCR and no-pCR patients. Our main result was that a higher α-diversity and a trend to higher richness were evident in patients achieving a pCR as compared with patients with residual disease after NACT. Moreover, at the species level, we observed a higher abundance of *B. eggerthii* in patients who achieve a pCR.

High α-diversity and richness of gut microbiome have been associated with response to treatment in several solid tumors. With regards to breast cancer, in a small cohort of patients of various cancer types, including 7 with BC, α-diversity before/during treatment (chemotherapy or immunotherapy) was significantly higher in responders as compared to non-­responders.^17^ Although different bacterial species have been linked to treatment response, no data on *B. eggerthii* in patients with BC emerged so far. Intriguingly, this species was associated with potential anti-inflammatory and anti-cancer effect via flavonoids metabolism in preclinical models,^18^ hence offering an interesting point to be further investigated. In a previous cohort of 32 patients with TNBC undergoing NACT, no difference in terms of α-diversity between patients who achieved a pCR and those with residual disease was found.^19^ However, some bacterial species were enriched in case of pCR. Another study of patients with TNBC including 30 women treated with NACT identified specific taxa that were abundant in patients with pCR; among patients with residual disease, those with partial response were enriched in Bacteroides caccae.^20^ Similarly, an analysis of microbiome of pre-NACT fecal samples in 85 subjects with newly diagnosed TNBC showed a prevalence of Bifidobacterium longum species in pCR patients.^21^ In a group of women with TNBC treated with NACT enrolled in the large CANTO study, LEfSe analysis showed a difference in microbiome composition of patients with positive nodes or residual disease after chemotherapy versus, respectively, those with post-treatment-negative nodes or pCR.^22^

Statistical comparison of longitudinal samples was limited by the small sample size, particularly considering that samples collected after antibiotics exposure were excluded, leaving only 7 patients with evaluable fecal samples for all 3 consecutive timepoints. This result highlights the limited feasibility of longitudinal monitoring of gut microbiome in this setting, mainly due to antibiotic exposure. However, with a descriptive intent, we looked at microbiome composition of matched samples before and after chemotherapy. In accordance with previous evidences in the same setting, no significant impact of chemotherapy on the overall bacterial composition over time was observed,^19^ as evaluated on fecal samples collected at t0 and t1. *B. eggerthii* persisted to be higher in pCR group, as observed at the baseline, and consistently with the stability of microbiome composition before and after chemotherapy. These findings deserve to be validated in a prospective time-variance study. Moreover, the evidence of no substantial change in gut microbiome structure after the exposure to cytotoxic treatments supports the rationale of assessing microbiome at the baseline, before chemotherapy. This latter data, together with the abovementioned ones highlighting the association of increased α-diversity and response to chemotherapy, suggest that an early intervention to modify the baseline microbiome could improve therapeutic outcome. In order to induce a modification in bacterial composition (i.e. by promoting the colonization of species potentially associated with response to anti-cancer therapies), a more direct approach should be considered. Probiotics, prebiotics, and dietary fibers favor gut microbial homeostasis^23,24^ and prevent dysbiosis. There are evidences that probiotics can exert anti-cancer activity by enhancing the immune system in preclinical studies in solid tumors.^25-28^ These supplements have been shown to reduce or prevent side-effects of anti-­cancer treatments.^29^ In this context, the manipulation of intestinal microbiome through probiotics and prebiotics, along with dietary intervention may represent a future perspective in cancer patients. Our study has limitations. First of all, the small sample size and the exploratory nature. Moreover, no complete information about factors that can influence microbiota composition, such as dietary habits and the presence of gastrointestinal symptoms, was available.^30^ Strengths of the study are: the enrollment of a consecutive cohort of patients treated at the same Institution, the homogeneity of therapies received, the conservative methodology, with the exclusion from statistical analysis of patients with a history of antibiotic treatment.

This first experience demonstrates that gut microbiome analysis in a population of early patients with TNBC undergoing NACT is feasible. Our findings give a hint about the role of microbiome composition in this population. Larger studies are needed in order to identify a microbiome signature with a potential impact on breast cancer prognosis and response to treatment. Gut microbiome actually represents a component of the immune system. In the last years, immunotherapy has become part of the clinical armamentarium in cancer therapy, and represents nowadays the standard of care for patients with TNBC in the neoadjuvant and first-line settings, in combination with chemotherapy. Our study was designed and conducted before the approval of neoadjuvant ICI for TNBC, thus we were not able to explore the relationship between microbiota and immunotherapy, and this should be recognized as a limitation. On the other hand, it is likely that specific subgroups of patients with TNBC (i.e. those with T<2 and N0 tumors or contraindications to ICI) will still be treated with chemotherapy alone in preoperative setting. However, given the leading role of immunotherapy in several solid tumors, it is of paramount importance to disentangle the complex triangulation between microbiome, immunity and cancer.

## Methods

### Patients

The MOON study was a prospective study conducted at the Istituto Oncologico Veneto IRCCS of Padova (Italy). Eligible patients were consecutive patients aged 18 years or older who had untreated, pathologically confirmed, triple-negative (defined as ER and PgR immunohistochemistry <1% and HER2 immunohistochemistry of 0+ or 1+, or if 2+, fluorescent in-situ hybridization [FISH] showing no amplification), non-metastatic, invasive BC candidate to neoadjuvant chemotherapy as per local clinical practice.

The study protocol was approved by the Institutional Ethical Committee. All patients provided written informed before chemotherapy start.

### Objectives

The primary endpoint was to determine the feasibility of longitudinal fecal samples collection in this patient population. Secondary endpoints were: i) to explore the association of gut microbiome composition with clinicopathologic characteristics; ii) to assess the impact of gut microbiome composition on the probability of achieving a pCR, and iii) to describe the effects of chemotherapy on longitudinal individual changes in gut microbiome composition.

### Fecal Samples Collection

Fecal samples were collected at 3 different timepoints (before chemotherapy start, t0; at 1 week from chemotherapy completion, t1; at 8 weeks from chemotherapy completion, t2) using specific vials (Shield Tube Zymo) containing a solution to inactivate pathogens. Of a total of 75 planned fecal samples (3 for each patient), 72 specimens were collected and stored at −80 °C until shipment to GenProbio srl, an academic spinoff of the University of Parma, under controlled temperature, for microbiome analysis.

### DNA Extraction and 16S rRNA Gene Amplicon Sequencing

DNA extraction from fecal samples, 16S rRNA gene sequencing and microbiome analysis were performed by GenProbio srl. DNA was extracted by using the QIAamp DNA Stool Mini kit following the manufacturer’s instructions (Qiagen Ltd, Strasse, Germany). 16S rRNA was amplified from extracted DNA using a primer pair Probio_Uni and/Probio_Rev (5ʹ-CCTACGGGRSGCAGCAG-3ʹ/5ʹ-ATTACCGCG­GCTGCT-3ʹ), targeting the V3 region of the 16S rRNA, and sequenced by using MiSeq (Illumina) at the DNA sequencing facility of GenProbio srl.^31^

### Bioinformatic Analysis

Forward and reverse reads were preprocessed and analyzed using the Quantitative Insights into Microbial Ecology pipeline (QIIME2, version 2020.8).^32^ As a first step, primer sequences removal was performed by means of cutadapt^33^ permitting no indels and an error rate equal to 0, requiring an overlap of 10 nucleotides, and allowing for wildcard read matching (--p-no-indels; --p-error-rate 0; --p-overlap 10; --p-match-read-wildcards). The reads in which no adapter sequence was found were discarded (--p-discard-untrimmed). After preprocessing, the amplicon sequence variant (ASV) table was constructed using a *de novo* approach using the DADA2^34^ bioinformatic plugin. The taxonomic assignment of each ASV was performed using the Greengenes database^35^ (version 13_8) and a Naive Bayes classifier trained on the target region selected for the present study (V3) to achieve a superior accuracy in taxonomic classification.

Alpha (Richness, Pielou, Shannon, Simpson indices) and beta (Bray-Curtis, Jaccard, Weighted and Unweighted Unifrac measures) diversity were calculated for microbial community diversity analysis applying a rarefaction level equal to 31613. This cutoff was chosen after verification (by means of a rarefaction plot) that it was placed after each rarefaction curve had reached its plateau. Additionally, beta diversity measures were used for ordination analysis with PCoA technique. Alpha diversity analysis was performed via QIIME2 dedicated plugins and graphically rendered in R (version 4.0.2), while beta diversity calculation and ordination plot production were performed in R using phyloseq (version 1.32.0) and vegan (version 2.5-7) packages. For the latter task, data were previously normalized using GMPR tool^36^ (version 0.1.3) to allow for robust comparison between samples.

### Tumor Samples Collection and Analysis

Tumor samples from the diagnostic core biopsy and from surgery were collected at Istituto Oncologico Veneto, Padua, Italy. pCR was defined as the absence of invasive residual disease in both the breast and axillary lymph nodes (ypT0/is, N0). TILs were centrally assessed on the diagnostic core-­biopsy and on the surgical specimen in case of residual infiltrating disease after neoadjuvant treatment. TILs were assessed following the TILs Working Group Recommendations.

### Statistical Analysis and Sample Size

The sample size of this exploratory study was calculated based on a feasibility endpoint, in order to demonstrate the practicability of longitudinal fecal samples collection at 3 timepoints for TNBC patients undergoing neoadjuvant chemotherapy. We aimed at obtaining an 80% rate of adequately collected fecal samples suitable for proper gut microbiome analysis (16s RNA sequencing). Considering a 95% CI with a margin error of ±9%, 75 samples to be collected from 25 patients were required.

In the present work, depending on the normality of analyzed data a *t* test or a Wilcoxon-Mann-Whitney test were applied to test for differences in alpha indices values between groups, as well as to test for differences in the Firmicutes/Bacteroidetes ratios between pCR and non-pCR groups at t0 and t1. The level of statistical significance was set at *P < .*05. A Dixon test was run before performing the tests to check for the presence of outlier values to exclude from the comparisons. The differences on beta diversity were tested using a PERMANOVA test applied on all the considered beta metrics. Finally, the differential abundance testing was performed using the *corncob* tool^37^ that implements a beta-binomial model specifically designed to deal with microbial abundance data.

## Supplementary Material

oyad060_suppl_Supplementary_Material

## Data Availability

Data will be made available upon specific request to the corresponding author Prof. Maria Vittoria Dieci (mariavittoria.dieci@unipd.it).
