# Supplementary material for: Characterization of Gut Microbiome Composition in Patients with Triple-Negative Breast Cancer Treated with Neoadjuvant Chemotherapy
Source: Oncologist. 2023 Mar 20;28(9):e703–11. doi: 10.1093/oncolo/oyad060 (PMC10485294; doi:10.1093/oncolo/oyad060)

**Supplementary Figure 1.** Consort diagram of the MOON study.

45 samples included in final analyses

♦ t0: 22

♦ t1: 14

♦ t2: 9

68 Samples passing pre-processing filters suitable for analysis

♦ t0: 23

♦ t1: 21

♦ t2: 24

4 samples not passing pre-processing filters

♦ t0: 2

♦ t1: 2

♦ t2: 0

23 samples collected within 90 days from last antibiotic therapy excluded from final analyses

♦ t0: 1

♦ t1: 7

♦ t2: 15

3 samples not collected

♦ t0: 0

♦ t1: 2

♦ t2: 1

72 samples collected

♦ t0: 25

♦ t1: 23

♦ t2: 24

75 planned samples

25 enrolled patients

**Supplementary Table 1.** P-values obtained from the Dixon test on alpha diversity values at ASV and species level, and related sample sizes. P-values under the threshold of 0.05 are highlighted in bold red. Abbreviations: ASV, amplicon sequence variant.

| Alpha: ASV level | pCR | Dixon test p-value | N |
| --- | --- | --- | --- |
| Shannon | Yes | 0.1866 | 7 |
|  | No | 0.746 | 8 |
| Pielou | Yes | 1 | 7 |
|  | No | 1 | 8 |
| Richness | Yes | **0.0199** | 7 |
|  | No | 0.9126 | 8 |
|  | | | |
| Alpha: Species level | pCR | Dixon test p-value | N |
| Shannon | Yes | 0.2267 | 7 |
|  | No | 1 | 8 |
| Pielou | Yes | 0.6049 | 7 |
|  | No | 0.0843 | 8 |
| Richness | Yes | **0.0203** | 7 |
|  | No | 0.1964 | 8 |

**Supplementary Table 2.** P-values obtained from the Dixon test on Firmicutes/Bacteroidetes ratio values, and related sample sizes. Abbreviations:F/B, Firmicutes/Bacteroidetes.

| F/B Ratio | pCR | Dixon test p-value | N |
| --- | --- | --- | --- |
| Baseline | Yes | 1 | 9 |
|  | No | 0.102 | 5 |
| Week1 | Yes | 1 | 9 |
|  | No | 1 | 5 |

w

**Supplementary Figure 2.** Box plot of baseline α-diversity between pCR and no-pCR patients at the species level according to Shannon Index (a), Pielou Eveness (b) and Richness (c). Outlier values are depicted in white and were excluded from the statistical test. Abbreviations: pCR, pathological complete response.

**Supplementary Figure 3.** Firmicutes/Bacteroidetes ratio in matched samples at t0 and t1 according to pCR. Abbreviations: pCR, pathological complete response.


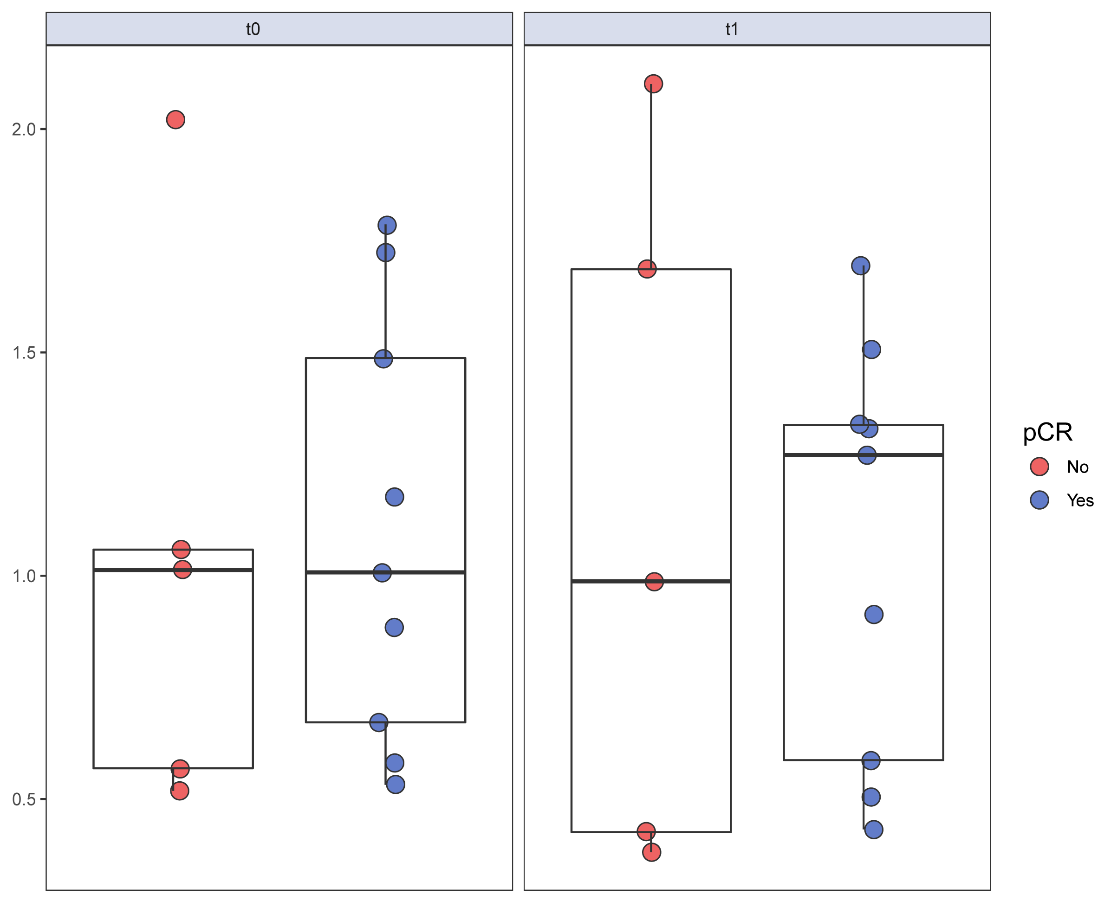

Supplement: oyad060_suppl_Supplementary_Material [file oyad060_suppl_supplementary_material.docx]
